# Batterer typologies: substance use, impulsivity and results of an IPVAW offender treatment program in Spain

**DOI:** 10.3389/fpsyt.2024.1492218

**Published:** 2025-01-30

**Authors:** Pedro V. Mateo-Fernández, Iria Osa-Subtil, Román Ronzón-Tirado, María Elena de la Peña Fernández

**Affiliations:** ^1^ Department of Psychology, Faculty of Biomedical and Health Sciences, European University of Madrid, Madrid, Spain; ^2^ Mental Health Research Group (MHeRG), Faculty of Medicine, Universidad Alfonso X el Sabio de Madrid, Madrid, Spain; ^3^ Department of Personality, Evaluation and Clinical Psychology, Faculty of Psychology, Universidad Complutense de Madrid, Madrid, Spain; ^4^ Department of Medicine, Faculty of Biomedical and Health Sciences, European University of Madrid, Madrid, Spain; ^5^ Department of Psychology, Faculty of Health Sciences, University of Deusto, Bilbao, Spain

**Keywords:** Intimate Partner Violence against Women (IPVAW), latent class analysis, substance use, impulsivity, batterer typology

## Abstract

**Introduction:**

Batterer impulsivity and substance use are relevant factors in the study of gender violence. Impulsivity is defined by the tendency to act suddenly and without forethought. Combined with drunkenness, it can materially increase the likelihood of intimate partner violence.

**Methods:**

The present study examines substance use and impulsivity among a sample of 243 men convicted of IPVAW offences under the Spanish Gender Violence Act (Organic Law 1/2004) in relation to the levels of violence and psychopathologies presented by these perpetrators, in order to understand the results of court-ordered psychological treatments provided under Spain’s Gender Violence Offenders Intervention Program. The participants, aged an average 39.1 years, were classified into three types based on demographic factors, substance use and other relevant variables. Meanwhile, the tools used included AUDIT and EuropASI to assess alcohol consumption, CTS2 to measure the frequency and intensity of violent behaviors over the last year, and SCID-II for personality disorders.

**Results:**

Our findings reflected marked improvements in conflict resolution strategies, especially in terms of reduced psychological violence and sexual coercion, but not physical violence. Impulsivity and early-onset alcohol use were identified as key risk factors for violent behavior. Latent class analysis revealed the existence of three sub-types, comprising high-risk batterers displaying high levels of aggression and drinking problems, low-risk batterers displaying high levels of secondary psychopathy, and medium-risk batterers.

**Discussion:**

The study underscores the need for differentiated treatment approaches to address both psychological problems and substance use, while highlighting the need for personalized interventions to rein in violent behavior and prevent reoffending. We We propose a future longitudinal study to throw light on the subsequent developmental paths taken by IPVAW offenders.

## Introduction

Intimate Partner Violence against Women (IPVAW) constitutes an egregious breach of basic human rights, requiring effective measures to protect women and children and advance toward a fairer, more equitable society (1). Violence against women may be psychological, coercive, physical and/or sexual (2, 3), and it is often associated with factors such as cognitive distortion, conflict resolution styles and personality variables.

The primary aim of this study is to throw light on the nature of substance use and impulsivity in different typologies of batterers based on levels of violence and the presence of psychopathologies among men convicted of gender violence offences in Spain. However, we also examine changes in pre- and post-treatment measures of aggression based on batterer profiles.

Efforts to understand intimate partner violence have driven the exploration of different batterer profiles. Despite the abiding consensus that no single, overarching batterer profile can be established (4), researchers have sought to identify the defining characteristics of men who commit IPVAW offences, and so distinguish those who perpetrate acts of violence from those who do not (5), and to identify the salient traits of actual abusers (6, 7).

Holtzworth-Munroe and Stuart (8) proposed a typology based on three dimensions, namely the severity of violence, the generality of violence and batterer psychopathologies. Based on their review of the existing literature, they were able to distinguish three subtypes of batterer based on three dimensions. These are “(a) *the severity of marital physical violence and related abuse, such as frequency of the violence and psychological and sexual abuse*; (b) *the generality of the violence* (i.e., family-only or extrafamilial violence) *and related variables such as criminal behavior and legal involvement*; and (c) *the batterer’s psychopathology or personality disorders*.” They further hypothesized that research applying these three descriptive dimensions would generally distinguish three main batterer subtypes, namely “(a) *family-only batterers*, (b) *borderline or dysphoric batterers* and (c) *generally violent/antisocial batterers”.* This typology has been validated in a range of different contexts (9), supporting the existence of different batterer subtypes each with their own distinctive characteristics.

The reality of intimate partner violence is complex and highly varied. Indeed, the very diversity of batterer characteristics itself suggests that they do not all form part of a single, uniform group. This heterogeneity has led to a growing awareness of the need to study psychopathological variables such as borderline personality traits (9), antisocial behaviors (10), drinking patterns (11, 12) and impulsivity (13, 14) as predictive factors for intimate partner violence.

Batterer impulsivity and substance abuse have now been recognized as key intensifiers of intimate partner abuse, driving both the frequency and severity of violent episodes (15). Substance-fueled disinhibition and loss of impulse control are associated with a significant increase in the likelihood of violent behaviors (16, 17). This is because substance use lowers the individual’s capacity to control his own emotions and actions, facilitating aggressive, impulsive responses in situations of conflict. Meanwhile, impulsivity defined as the tendency to act suddenly without any thought for the consequences only exacerbates violent responses, especially under conditions of stress (18).

It has recently been suggested that new IPVAW offender typologies may be needed to address the risk of violent incidents and the likelihood of recidivism (19), given that impulsivity can trigger physical and emotional violence and is associated with a high probability of repeat incidents, heightening the risk of increasingly devastating outcomes (20). These expanded typologies are differentiated based on the level of risk and provide a more detailed framework within which to understand intimate partner violence and seek solutions to what is a multifaceted problem (6), in particular as regards the risk of violent outbursts and repeat incidents. For example, Cavanaugh and Gelles (19) proposed three differentiated groups of abusers based on the frequency and severity of violence, and the presence of psychopathologies and prior criminal histories. These comprise (a) a *low-risk* group, consisting of offenders who were rarely violent, had committed less severe acts of IPVAW and did not usually present significant psychopathologies or criminal records; (b) a *medium-risk* group of habitually violent offenders responsible for relatively mild acts of aggression, who were likely to present moderate to high levels of psychological disorder; and (c) a *high-risk* group of frequently violent offenders responsible for acts of serious abuse, presenting a range of psychopathologies and a material criminal record. Even so, existing profiles are still unable fully to describe the heterogeneity of batterers or the relationship between offending and known risk variables such as substance use and impulsivity. Further insight will therefore be crucial to the development of effective, specific interventions to address the needs and risks associated with each subtype of IPVAW offender.

In light of the foregoing, the present study seeks to throw light on the characteristics of substance use and impulsivity among a sample of men convicted of IPVAW offences under the Spanish Gender Violence Act (Organic Law 1/2004) in relation to the levels of violence and psychopathologies presented by these perpetrators. The criteria used in the study to establish the batterer typologies using latent class analysis comprised the frequency and severity of episodes of intimate partner violence, general levels of violence and other associated IPVAW risk factors, such as personality profiles, alcohol abuse, impulsivity and the presence of emotions linked to violent outbursts.

## Method

### Ethics declaration

This study was approved by the Complutense University of Madrid’s Faculty of Psychology Academic Ethics Board on June 7, 2021. The approximate duration of treatments and the purpose and procedures employed in our research were explained to all participants, and their informed consent was obtained in all cases.

### Participants

All of the men participating in this study had been convicted of gender violence offences subject to mandatory enrolment in a special program under the oversight of the Spanish courts. The participants had therefore been ordered to follow a course of psychological treatment rather than serving a custodial sentence of less than two years, pursuant to Part IV of the aforementioned Spanish Gender Violence Act, 2004.

The total study sample consisted of 243 men ranging in age between 20 and 80 years (average age = 39.10; SD: 11.1). In terms of academic attainment, 53.90% (n=110) of participants had completed secondary and 23.0% (n=47) primary level education. A further 18.10% (n=37) had attended a university and 4.90% (n=10) had no formal qualifications. In terms of socioeconomic status, meanwhile, 61.90% (n=117) of the sample were classified as middle class and 19.60% (n=37) as lower class. Upper-middle class participants made up 12.20% (n=23) of the participants and 6.30% were upper class. By nationality, the participants were 68.30% (n=157) Spanish, 18.30% (n=42) South American and 8.70% (n=20) from other European countries. Participants of African origin made up 3.90% (n=9) and 0.90% (n=2) were classified as from the “Rest of the World”. Finally, 22.20% (n=40) of participants were married or had a stable partner, 27.70% (n=50) were separated or divorced, and 48.30% (n=87) were single.

### Procedure

The participants were enrolled in the *Gender Violence Offenders Intervention Program – Alternative Measures* (PRIA-MA) set up under the aegis of the Spanish Department of Penitentiary Institutions (21), which consists of three phases – *assessment, intervention* and *tracking.* In the first phase, the participants were assessed on all items included in the Measures sections of the self-report questionnaires applied to establish an individual pre-treatment baseline in each case. The intervention phase, meanwhile, comprised 32 weekly sessions lasting two hours each spread over 10 modules. Participants were provided with the informed consent forms in the first session, when the details of the study were explained and all concerns voiced were addressed, including the rules of the program and the reasons why the individual concerned had been included. Upon completing the program, each participant was subjected to a post-treatment assessment using the same questionnaires as applied in the pre-treatment phase in order to re-evaluate the issues addressed. The program ended with the tracking phase, which consists of a final session basically to allow for the clarification of participants’ concerns and to review and assess their future plans in order to guarantee lasting results and alignment of the intervention with their future needs.

### Measures

#### Sociodemographic questionnaire

The questionnaire was used to obtain data on the participants’ sociodemographic and personal variables, including age, academic attainment, social class, marital status, nationality and occupation.

#### Severity and frequency of intimate partner violence

These variables were measured using the Revised Conflict Tactics Scale (CTS2; 22; Spanish adaptation by 23), a self-report questionnaire comprising 78 items (39 for perpetration and 39 for victimization) referring to the last year of the subject’s relationship. It consists of 5 subscales covering negotiation, psychological violence, physical violence, injury and sexual coercion. According to the scale’s authors, the alpha coefficient varied between 0.79 and 0.95. The scales for minor psychological violence (α=0.80), minor physical violence (α=0.59) and minor sexual coercion were obtained in this study. No α was obtained in the latter case because the results did not vary sufficiently.

#### Substance use

Two tools were used to measure substance use and dependence. Alcohol-related disorders were measured using AUDIT (24–26), and module III of the European Addiction Severity Index (EuropASI) was used to measure consumption of alcohol and drugs (27, 28; Spanish adaptation by 29). The former test, which consists of 10 items, addresses issues related with drinking habits (Direct Score, DS≥9), alcohol dependence (DS≥21) and related outcomes. The test has an internal consistency of α = 0.80 and displays excellent sensitivity and specificity (30). The internal consistency of the test in the present study was α = 0.86. EuropASI consists of a semi-structured clinical interview covering 141 items exploring eight aspects of the dependent individual’s circumstances, a factor which may influence the emergence of substance abuse problems, including a module for alcohol and drug use comprising 28 items to assess consumption of both liquor and other narcotics (heroin, cocaine, amphetamines and cannabis). In addition to establishing individual levels of alcohol consumption, this tool was used to classify all other substances as Central Nervous System (CNS) stimulants or depressants.

#### Borderline and antisocial personality traits

The Self-Report Assessment of the DSM-IV-R Personality Disorders (SCID-II; 31) was used alongside the Borderline Personality Organization Scale (BPO Scale; 32) to measure both dimensions. Thirty items from the SCID-II borderline personality disorder (BPD) and antisocial personality disorder (APD) scales were used (15 from each with a cut-off threshold of DS≥5 in both cases), because they are both significantly associated with batterers. The original study found a test-retest reliability of 0.84 for antisocial disorder and 0.37 for borderline symptoms. In this study, the tool obtained confidence values of α = 0.89 for both scales.

#### Psychopathy

The secondary psychopathy subscale of the Levenson Self-Report Psychopathy Scale (LSRP; 33) was used in view of its tried-and-tested psychometric properties (34, 35). This self-report tool contains 26 items addressing issues related with manipulative behaviors, insensitivity and egotistical attitudes (*primary psychopathy;* DS≥20) and antisocial and impulsive behaviors (*secondary psychopathy;* DS≥20). In terms of reliability in the present study, the LSRP scored an alpha of 0.76 on both scales.

#### General violence and violent emotions

These variables were measured using the Buss-Perry Aggression Questionnaire (AQ; 36; Spanish adaptation by 37) and the State-Trait Anger Expression Inventory (STAXI-2; 38; Spanish adaptation by 39). The AQ contains 29 items distributed across 4 subscales (physical aggression, verbal aggression, anger and hostility). The psychometric properties of the Spanish adaptation of this tool were α = 0.86 for physical aggression, α = 0.68 for verbal aggression, α = 0.77 for anger, and α = 0.79 for hostility. Meanwhile, the scores obtained in the present study were α = 0.81 for physical aggression, α = 0.52 for verbal aggression, α = 0.72 for anger, and α = 0.81 for hostility. STAXI-2 comprises 49 items for both state anger (DS≥21) and trait anger (DS≥18), as well as the different ways in which subjects express and control these feelings. The test scored well in terms of internal consistency, presenting values that ranged from α = 0.89 for state anger to α = 0.64 for the expression of anger in the Spanish adaptation.

#### Impulsivity

Traits associated with impulsivity were assessed using Plutchik’s Impulsivity Scale (40; Spanish validation by 41). This tool contains 15 items measuring the impulsiveness of a subject’s actions (DS≥20) distributed in 4 subscales (ability to plan ahead, control of emotional states, control of eating, spending and sexual relations, and control of other behaviors). Prior studies scored α = 0.74 on internal consistency, but a value of α = 0.73 was obtained for the scale as a whole in this study.

### Analysis

We began by performing a descriptive analysis of the variables used in the study to evaluate their distributive properties, including estimates of centrality and dispersion. We then preceded with a Latent Class Analysis (LCA) to discern underlying structures within the data set (see variables in **Table 1**). This methodological approach, which is anchored in probabilistic principles, facilitated classification of the subjects into homogeneous segments based on observed response patterns, allowing precise identification of latent profiles for violent behaviors. A Repeated Measures Analysis of Variance (ANOVA) was then performed to test the frequency of low-level violence (psychological and physical aggression, and sexual coercion) before and after the intervention, treating the latent classes identified as a secondary factor. This analysis was based on the premise that intra-subject variability in the repeated measures can be explained in part by the classification of the different latent classes identified. We verified the assumptions of normality and homoscedasticity. Finally, we performed descriptive analyses to determine patterns of substance use based on the relative frequency of the behaviors concerned.

**Table 1:** Descriptive statistics obtained from measurement tools.

Variable	% (n)
General Aggression	
AQ-Physical aggression	31.70% (77)
AQ-Verbal aggression	14.00% (34)
AQ-Anger	25.90% (63)
AQ-Hostility	38.30% (93)
Alcohol use	
AUDIT	19.30% (47)
Psychopathy	
Levenson-secondary	84.40% (205)
Personality	
SCID II-Borderline	38.30% (93)
SCID II-Antisocial	13.60% (33)
Impulsiveness	
Plutchik	8.20% (20)
Anger	
STAXI-Trait anger	26.70% (65)
STAXI- Anger expression and control	17.70% (43)

From the standpoint of interpretation, we selected the models that best represented the data based on the inherent loss of statistical metrics. To this end we used the loss of verisimilitude in conjunction with the Akaike information criterion (AIC; 42), the conditional Akaike information criterion (CAIC; 43), the Bayesian information criterion (BIC; 44) and its sample-size adjusted variant (SABIC; 44, 45) to select the best 6 models in the LCA (1 to 6 classes). Graña et al. (6) provided the theoretical criterion. Meanwhile, we used entropy as the subject classification accuracy indicator, taking scores above 0.8 to show robust class assignation (46). Variables were dichotomized as absent or present based on the criteria obtained from the validation studies for each scale, choosing the highest term in each case. The statistical analyses were run on R (version 4.2.3) using the RStudio interface.

## Results

### Descriptive statistics

The descriptive results of the study (see **Table 1**) showed that hostility (AQ) was the commonest measure of aggression, closely followed by physical aggression (AQ). In terms of associated problems, meanwhile, we found a high proportion of subjects with secondary psychopathy in the sample, as well as moderate levels of trait anger and the presence of borderline personality disorders and low levels of antisocial disorder, alcohol use and expression of anger.

The results for pre- and post-treatment violence (CTS) reflected initially lower scores for sexual coercion (M=0.80; SD=5.99) followed by physical aggression (M=2.20; SD=6.09), and higher scores in the sample for psychological aggression (M=8.49; SD=17.30). The lowest level of post-treatment aggression was again found in relation to sexual coercion (M=0.57; SD=3.85), while the physical aggression variable displayed similar mean levels although with higher variability than in the pre-treatment score (M=2.45; SD=11.90). The higher post-treatment aggression scores observed were also found to be present in the case of psychological aggression (M=5.97; SD=13.40).

### Latent class analysis

Five latent class models including between 2 and 6 classes were estimated and compared to determine the structure with the best fit to our data from both the statistical and theoretical standpoints. Based on our evaluation of the fit indices for the model data (see **Table 2**), the three-class model appears to display the best balance in terms of statistical and conceptual fit. This finding is supported by the lower BIC, SABIC and CAIC scores obtained for this model, revealing that the model presents a better fit than the alternatives, whether containing more or less classes. Furthermore, the entropy value of this model is adequate (>0.80) and its smallest class makes up 27% of the sample, preventing problems with very small or unrepresentative classes

**Table 2:** Fit parameters of the 2- to 6-class models.

Number of classes	LogLik	BIC	SABIC	AIC	CAIC	Entropy	Smallest class size (%)
2	-1249	2625	2552	2545	2648	0.75	49.00%
3	-1192	2577	2466	2455	2612	0.80	27.00%
4	-1180	2618	2469	2454	2665	0.82	11.00%
5	-1154	2633	2446	2427	2692	0.83	2.00%
6	-1143	2676	2451	2428	2747	0.85	6.20%

LogLik, Log-likelihood; BIC, Bayesian Information Criterion; SABIC, Sample-size Adjusted Bayesian Information Criterion; AIC, Akaike Information Criterion, CAIC, Conditional Akaike Information Criterion.

The entropy value of the three-class model (0.80) is significant, insofar as it measures the accuracy of the classification of individuals into latent classes. An entropy value above 0.80 is considered a critical threshold, as it ensures that class assignments are clear and well-defined, which improves the validity of the model. Although the four-class model has a slightly higher entropy (0.82), it was decided not to opt for this because its smallest class represents only 11% of the sample, which could compromise its theoretical relevance and representativeness compared to the three-class model, whose smallest class comprises 27% of the sample.

In conceptual terms, the three-class structure offers a better fit both statistically and theoretically, as it more accurately reflects the variability present in our data without becoming over-specified, as occurs in the four- or five-class model, where the smaller classes are likely to be unrepresentative or overly fragmented. In contrast, the two-class model oversimplifies the variability of the data, failing adequately to capture the complexity of the phenomenon analyzed. The three-class structure, then, allows for a more coherent and meaningful classification, with theoretical implications that point to the existence of well-differentiated subgroups within the sample.

Class 3 stood out as presenting the highest probability of violence, specifically in the form of physical aggression (83.40%), verbal aggression (44.71%), irascibility (91.00%) and hostility (83.00%). In comparison with the other classes, the Class 3 participants also display an intermediate probability of presenting serious problems of alcohol abuse (24.00%), borderline personality disorder (BPD 51.10%) and antisocial personality disorder (APD 15.00%), and high levels of trait anger (40.00%), expression of anger (18.00%) and impulsivity (14.00%) However, this class also displays the lowest scores for the secondary psychopathy variable with a 77.80% probability.

Meanwhile, Class 1 scores significantly lower on physical aggression (10.80% probability) and the likelihood of verbal aggression is nugatory, making this the least violent group in these respects. The levels of irascibility (5.04%) and hostility (35.40%) are also low in this class. However, its members display a high probability of secondary psychopathy (86.00%). This class also presents the highest probability of BPD (93.10%) and APD (46.63%), indicating the prevalence of these disorders among participants. These subjects are also the most likely to present impulsivity (18.00%), trait anger (52.70%) and expressions of anger (38.95%).

Class 2 presents a lower probability of physical and verbal aggression (14.30% and 4.52, respectively) than Class 3, but a higher probability than Class 1 in both cases, placing this group on an intermediate level of aggression. The scores for irascibility (5.14%) and hostility (21.80%) are similar. Meanwhile, the 86% probability of secondary psychopathy is the highest of any class, but the probability of BPD (13.80%) and APD (0.88%) are the lowest, suggesting a more stable psychological profile. Impulsivity also scores extremely low (0.69%), as do trait anger (11.80%) and expression of anger (9.43%), reflecting the lowest tendency of any class to engage in impulsive behaviors and express anger.

Finally, **Table 3** below summarizes the salient characteristics of the three IPVAW offender typologies considered in this study.

**Table 3:** Characteristics of IPVAW typologies.

Typologies	Characteristics
Type I: High Risk Violent Offender (Class 3)	Higher values for aggression, irascibility and hostility. Below-the-mean levels of substance use, psychopathologies and factors associated with anger (trait and expression).
Type II: Offender presenting moderate-risk psychopathologies (Class 1)	Higher levels of substance consumption and psychopathologies (impulsive, borderline and marked antisocial traits) with significant anger components (trait and expression).
Type III: Low-risk offender (Class 2)	Low values for physical and verbal aggression Lower levels of irascibility, hostility, habitual alcohol and other substance use, borderline and antisocial personality characteristics, impulsivity, and factors associated with anger (trait and expression).

Prepared by the authors.

### Changes in the treatment of each class and types of substance use

With regard to treatment outcomes relating to each of the three types of violence (psychological and physical violence and sexual coercion), we observed a significant drop in the frequency of episodes of psychological aggression following treatment (see **Table 4**). However, this finding was significant only in Class 1 compared to Class 2 with a difference of 8.41 points in the mean score for the former compared to 1.07 for the latter. Statistically changes in frequency were also observed in the case of sexual coercion in Classes 1 and 2, but not in the others. The difference found in Class 1 was 1.68 points and 0.07 in Class 2, and in the latter case the score obtained actually worsened after treatment. No material differences were found in the frequency of incidents involving physical violence before and after treatment.

**Table 4:** Mean values and changes in the frequency of violent behaviors before and after treatment by class.

	M sample (n=243)		F	M Class 1 (n=47)		M Class 2 (n=135)		M Class 3 (n=61)		F	Bonferroni
	Pre	Post		Pre	Post	Pre	Post	Pre	Post		
Psychological violence	8.42	4.83	14.61***	13.30	4.89	4.82	3.75	7.15	5.18	5.68***	1-2*** 1-3 2-3
Physical violence	2.19	1.82	0.52	4.38	2.70	0.65	1.14	1.15	1.62	1.64	1-2*** 1-3*** 2-3
Sexual Coercion	1.09	0.450	6.87***	2.12	0.44	0.104	0.17	1.04	0.38	4.45**	1-2** 1-3 2-3

**p <.01; ***p < .05.

Turning to the sociodemographic variables (see **Table 5**), the distribution of average age was found to be fairly homogeneous across the different classes with a mean age of 38.0 in Classes 1 and 3, and 40.5 years in Class 2. The majority nationality in all classes was Spanish, accounting for 75.00% of the participants included in Class 3, 69.00% in Class 2 and 56.80% in Class 1. In the case of educational attainment, meanwhile, Class 3 included the highest percentage of participants with only primary level qualifications (32.00%), while Class 2 had the highest percentage of individuals with university studies (23.50%). Finally, Class 1 stands out in terms of marital status with the highest percentage of single men at 60.60% compared to 45.10% in Class 2 and 48.90% in Class 3.

**Table 5:** Sociodemographic and consumption variables for each class.

	Type II (Class 1)	Type III (Class 2)	Type I (Class 3)
M (SD)			
Age	38.00 (12.15)	40.50 (11.29)	38.00 (9.49)
% (n)			
Nationality			
- Spanish	56.80% (25)	69.00% (87)	75.00% (45)
- Rest of Europe	6.80% (3)	10.30% (13)	6.70% (4)
- Latin America	27.30% (12)	15.90% (20)	16.70% (10)
- Africa - Rest of the world	9.10% (4) 0.00% (0)	3.20% (4) 1.60% (2)	1.70% (1) 0.00% (0)
Level of education			
- Primary	17.90% (7)	20.90% (24)	32.00% (16)
- Secondary	62.90% (27)	48.70% (56)	54.00% (27)
- University students	10.30% (4)	23.50% (27)	12.00% (6)
- No education	2.60% (1)	7.00% (8)	2.00% (1)
Marital status			
- Married/partnered	12.10% (4)	25.50% (26)	26.70% (12)
- Separated/divorced	27.30% (9)	29.40% (30)	24.40% (11)
- Single	60.60% (20)	45.10% (46)	48.90% (22)
Lifetime alcohol consumption	50.00% (23)	30.37% (41)	26.67% (16)
Lifetime use of stimulants	30.43% (14)	11.85% (16)	20.00% (12)
Lifetime use of other depressant substances	52.17% (24)	21.48% (29)	33.33% (20)
Alcohol consumption in the last month	41.30% (19)	15.56% (21)	16.67% (10)
Stimulant use in the last month	8.70% (4)	5.19% (7)	11.67% (7)
Use of other depressant substances in the past month	52.17% (24)	21.48% (29)	20.00% (12)

The substance use variable revealed that 50.0% of the participants classified in Class 1 had consumed alcohol at some time in their lives, compared to 30.37% in Group 2 and 26.67% in Class 3. However, the consumption of alcohol in the last month was markedly lower in Class 2 (15.56%) compared to either Class 1 (41.30%) or Class 3 (16.67%). Meanwhile, the use of stimulants was highest in Class 1 both over participants’ lifetimes (30.43%) and in the last month (8.70%). Class 1 also presents greater use of depressant narcotics than the other Groups both over the participants’ lifetimes (52.17%) and in the last month (52.17%)

## Discussion

This study of impulsivity and substance use among convicted intimate partner violence (IPVAW) offenders reveals that subjects belonging to one of the three typologies of batterers scored better on measures of violence after treatment for both aggression and substance-use patterns. The Latent Class Analysis performed allowed identification of three subtypes of batterers among a sample of men convicted of gender violence offences in Spain, in line with previous research (6, 8, 19, 47).

### Batterer typology and risk factors

Our results support the proposal that batterers can be classified into different subtypes, each with its own definitive characteristics that in turn need to be addressed in the design of interventions. This heterogeneity of batterer profiles is consistent with the existing literature, and it underscores the importance of adopting a differentiated approach to treatment and the prevention of intimate partner violence.

Impulsivity and early-onset drinking emerge as key risk factors for aggression, which is again consistent with the existing literature (48, 49). Defined by a tendency to act suddenly without considering the possible consequences, impulsivity may exacerbate violent responses, especially in situations of conflict or stress. This finding is in line with previous studies, which have identified impulsivity as a robust predictor of intimate partner violence (14, 20).

Meanwhile, early-onset alcohol use can have a long-term impact on neurologic development and on an individual’s capacity for emotional control, thereby heightening the risk of violent behaviors in intimate relations. This finding is consistent with other studies, which have shown a strong association between alcohol use and the perpetration of intimate partner violence (11, 12).

Importantly, the relationship between these risks factors and intimate partner violence is neither straightforwardly linear nor causal. Rather, there seems to be a complex interaction between impulsivity, substance use and other contextual and psychological factors involved in violent behavior. This complexity demands the adoption of multidimensional approaches to the prevention and treatment of intimate partner violence.

### Treatment results

Our assessment of the PRIA-MA program showed promising results in relation to the use of more adaptive strategies to conflict resolution. This approach was found to produce a significant reduction in psychological violence across the batterer subtypes after treatment, despite marked differences between the different classes. These findings are encouraging and suggest that the program is an effective means of addressing kinds of violence that are often more subtle and difficult to detect but can nevertheless have profound and lasting effects on victims.

The reduction in psychological violence is particularly significant insofar as abuse of this nature often precedes and accompanies more acute manifestations of physical violence. The program’s success in mitigating behaviors of this kind suggests that it effectively addresses the underlying thought patterns and attitudes contributing to intimate partner violence.

Another important finding was the reduction in episodes of sexual coercion, a form of violence that is all too often underrepresented in reports though it can have grave consequences for the mental and physical wellbeing of victims. Once again, the program’s success in mitigating such behaviors suggests that it effectively addresses beliefs and attitudes related with consent and mutual respect in intimate relations.

Notwithstanding these favorable outcomes, we did not find significant changes in physical violence, suggesting a need to improve the response to this issue in future interventions. Various factors may explain this failure to alter patterns of physical violence. To begin with, it suggests that physically violent behaviors are more change-resistant and may require more intensive or prolonged interventions. Meanwhile, it may also reflect a floor effect if the level of physical violence was already relatively low at the start of treatment, leaving little room for any further improvement.

These findings are consistent with previous studies addressing the results of treatment programs designed to reduce violence and curtail repeat episodes (50, 51). However, the variability observed in subjects’ responses to treatment across the different batterer types underscores the need to develop personalized interventions.

Importantly, the results of treatment appear to vary depending on the typology of the batterer. High-risk aggressors (Class 3) displayed greater improvements than those classified in the other groups, suggesting that the PRIA-MA program could be particularly effective in the most severe cases of intimate partner violence. This finding has profound implications for the allocation of resources and the intensity of treatment, if high-risk batterers can in fact benefit from more intensive interventions.

### Substance use patterns

Our analysis of substance use patterns revealed material differences between the batterer typologies. Class 1 (Type II), defined as users with medium-risk psychopathologies, displayed high levels of consumption both of alcohol and other substances. This finding suggests that there is a strong correlation between substance use and the presence of impulsive and antisocial psychopathologies (52).

Of particular concern was the high level of alcohol use found among Class 1 participants, 50.0% of whom claimed to have consumed liquor at some time in their lives and 41.30% admitted to doing so in the last month. These levels of consumption not only heighten the risk of intimate partner violence but may also exacerbate existing mental health problems and hamper treatment. Furthermore, consumption of stimulants and depressants in significant quantities among the members of this group suggest a pattern of polyconsumption, further complicating existing clinical symptoms and undermining therapeutic efforts.

A number of psychological and motivational factors may explain the varied substance consumption found in all of the batterer typologies. The individuals included in Class 1 may resort to drugs as a means of handling out-of-control impulses and emotions (53). This pattern of substance use may, then, represent an attempt to self-medicate as a defense against the symptoms of personality disorders and problems of emotional control.

Meanwhile, high-risk batterers (Class 3) may be driven more by the need for control and domination than by impulse, which would explain their lower levels of substance use (54). This finding further suggests that violence may be more instrumental and less reactive in this group. If true, this would have major implications for the design of interventions.

Low-risk batterers (Class 2) displayed lower levels of alcohol and drug use, suggesting that violent behaviors among these individuals are associated with situational factors and/or poor communication and conflict resolution skills rather than substance abuse or severe psychopathologies.

These findings underscore the importance of integrating the treatment of substance abuse into batterer intervention programs, especially in the case of Class 1 subjects. It also suggests that interventions should be tailored to the specific needs of each batterer typology, so as to address not only substance use but also the underlying psychological and contextual factors contributing to violent outbursts.

### Implications in practice

The findings of this study have important implications for both clinical practice and public policy design in the area of IPVAW prevention and treatment. In the first place, they suggest the need to develop differentiated treatment programs addressing both psychological problems and substance use. This kind of combined approach is important to tackle the complex factors involved in intimate partner violence.

In the case of Class 1 batterers displaying high levels of substance use and psychopathologies, it would be helpful to establish a twin-track treatment approach to address both substance abuse and mental health problems in tandem. Such an approach could include specific cognitive-behavioral therapies to help subjects rein in impulsivity and control their emotions, combined with interventions to mitigate drinking and drug-taking.

Interventions targeting high-risk batterers (Class 3) displaying high levels of violence but lower levels of substance use should focus rather on the beliefs and attitudes that uphold the edifice of controlling and dominant behaviors. This might include more intensive work on gender norms, equal relations and empathy-building.

In contrast, low-risk batterers (Class 2) could benefit more from interventions focused on the development of communication and conflict-resolution skills and on stress management. A more educational and prevention-oriented approach may be better suited to this group.

Our results also underscore the importance of implementing exhaustive evaluation procedures to identify individual offenders’ typologies and adapt interventions accordingly. This would mean developing and validating assessment tools to allow effective classification of batterers into the three typologies identified in this study.

Furthermore, our findings highlight the need for tight collaboration between the mental health services, addiction treatment practitioners and the providers of batterer intervention programs. Such interdisciplinary cooperation will be essential if we are to offer integrated treatments to address all of the factors concerned in intimate partner violence.

Finally, our findings have implications for the training of professionals working in the field. It is crucial to train therapeutic and other practitioners in the skills they need to recognize and address the diversity of batterer profiles, and to manage the comorbid problems of substance abuse and mental health issues that so often accompany intimate partner violence.

### Limitations and future directions

We need to recognize the limitations of this study in order adequately to contextualize our findings and point the way for future research. In the first place, the sample may suffer from selection bias, as it comprises exclusively men convicted of gender violence offences enrolled in a mandatory treatment program. This limits the generalizability of our findings to other populations, such as batterers who have remained undetected by the authorities and those who voluntarily seek treatment (4).

Meanwhile, the use of self-report tools to measure violence, substance use and other variables can result in bias due to social desirability issues, insofar as participants may understate violent behavior or substance use in order to present themselves in a better light (2). In these circumstances, it will be important to draw on multiple information sources in future studies.

A further limitation is the cross-sectional nature of the study, which prevents us from establishing any kind of causal relations between the variables considered. While we have identified associations between the batterer typologies, substance use and treatment responses, we are unable to infer any kind of causal link based on our data (9).

We suggest the following avenues for future research in order to address the limitations described and shed further light on the phenomenon of intimate partner violence:

1. Prospective longitudinal studies to identify more accurately the developmental routes taken by the different batterer subtypes. These studies could start in adolescence, or even childhood, and would examine the ways in which factors like exposure to violence, early-onset substance use and affective patterns contribute to the development of batterer profiles (55).
2. Use of multiple information sources, including victim and witness data and official registers. This would provide a fuller, more objective picture of the patterns of violence in question and would help overcome the limitations inherent in self-report tools (56).
3. Examination of the stability of the typologies identified over time and in different cultural contexts. This would help determine whether the typologies are universal or vary depending on the sociocultural context (6).
4. In-depth investigation of interactions between substance use, impulsivity and other risk factors involved in intimate partner violence. This could include experimental studies to examine how acute alcohol consumption affects impulsivity and aggression among the different batterer typologies (11, 12).
5. Assessment of the results of personalized interventions based on the typologies identified in this study. This could help with the development and evaluation of treatment programs aligned with the specific needs of each type of batterer (19).
6. Exploration of the role of self-preservation behaviors and resilience as factors that could moderate relationships between risk factors and the perpetration of intimate partner violence. This could provide valuable information for the development of future preventive interventions (48).
7. Investigation of long-term post-treatment paths, including recidivism rates and factors associated with the persistence of behavioral changes (50, 51).

## Conclusion

This study makes a significant contribution to our understanding of male batterer typologies and their association with substance abuse and impulsivity. The identification of three different batterer subtypes, each with its own distinct characteristics in terms of the patterns of violence, substance use and treatment response offers a solid basis for the development of more effective, tailored interventions. Our findings underscore the importance of addressing the heterogeneity of batterers in the design of treatment programs and prevention policies. The differential results of the PRIA-MA program depending on the different batterer typologies suggest that a one-size-fits-all approach may not be optimal, and that interventions tailored to the specific needs of each subtype could significantly improve outcomes. Furthermore, our findings in relation to substance use, impulsivity and intimate partner violence underscore the need to address these factors on an integrated basis in intervention programs. The integration of treatments for substance use and the management of impulsivity in batterer programs could improve their overall efficacy. This study also provides an empirical basis for the improvement of risk assessment procedures, allowing professionals more precisely to identify high-risk batterers who may need more intensive intervention measures or closer supervision. Despite the limitations described above, the results of this study open up new avenues for future research and have important implications for clinical practice and the design of public policy. Future studies addressing these limitations and/or exploring the proposed research paths will be crucial to progress in this field and, in the final analysis, to the mitigation of intimate partner violence and its impacts. In short, this study represents an important step toward a more nuanced and complete understanding of the phenomenon of intimate partner violence, providing valuable insight for the design of more effective prevention and intervention strategies aligned with the specific needs of different types of batterers.

## Data Availability

The datasets presented in this article are not readily available due to ethical and data privacy. Requests to access the datasets should be directed to pedrovmf@cop.es.
